# Next-generation sequencing of host genetics risk factors associated with COVID-19 severity and long-COVID in Colombian population

**DOI:** 10.1038/s41598-024-57982-3

**Published:** 2024-04-11

**Authors:** Mariana Angulo-Aguado, Juan Camilo Carrillo-Martinez, Nora Constanza Contreras-Bravo, Adrien Morel, Katherine Parra-Abaunza, William Usaquén, Dora Janeth Fonseca-Mendoza, Oscar Ortega-Recalde

**Affiliations:** 1 School of Medicine and Health Sciences, Center for Research in Genetics and Genomics (CIGGUR), Institute of Translational Medicine (IMT), Universidad Del Rosario, Bogotá, D.C Colombia; 2 Hospital Universitario Mayor - Méderi - Universidad del Rosario, Bogotá, D.C Colombia; 3 Populations Genetics and Identification Group, Institute of Genetics, Universidad Nacional de Colombia, Bogotá, D.C Colombia; 4 Departamento de Morfología, Facultad de Medicina e Instituto de Genética, Universidad Nacional de Colombia, Bogotá, D.C Colombia

**Keywords:** COVID-19, Long-COVID, Next-generation sequencing, Predictive model, Personalized medicine, Genetics research, Predictive markers

## Abstract

Coronavirus disease 2019 (COVID-19) was considered a major public health burden worldwide. Multiple studies have shown that susceptibility to severe infections and the development of long-term symptoms is significantly influenced by viral and host factors. These findings have highlighted the potential of host genetic markers to identify high-risk individuals and develop target interventions to reduce morbimortality. Despite its importance, genetic host factors remain largely understudied in Latin-American populations. Using a case–control design and a custom next-generation sequencing (NGS) panel encompassing 81 genetic variants and 74 genes previously associated with COVID-19 severity and long-COVID, we analyzed 56 individuals with asymptomatic or mild COVID-19 and 56 severe and critical cases. In agreement with previous studies, our results support the association between several clinical variables, including male sex, obesity and common symptoms like cough and dyspnea, and severe COVID-19. Remarkably, thirteen genetic variants showed an association with COVID-19 severity. Among these variants, rs11385942 (*p* < 0.01; OR = 10.88; 95% CI = 1.36–86.51) located in the *LZTFL1* gene, and rs35775079 (*p* = 0.02; OR = 8.53; 95% CI = 1.05–69.45) located in *CCR3* showed the strongest associations. Various respiratory and systemic symptoms, along with the rs8178521 variant (*p* < 0.01; OR = 2.51; 95% CI = 1.27–4.94) in the *IL10RB* gene, were significantly associated with the presence of long-COVID. The results of the predictive model comparison showed that the mixed model, which incorporates genetic and non-genetic variables, outperforms clinical and genetic models. To our knowledge, this is the first study in Colombia and Latin-America proposing a predictive model for COVID-19 severity and long-COVID based on genomic analysis. Our study highlights the usefulness of genomic approaches to studying host genetic risk factors in specific populations. The methodology used allowed us to validate several genetic variants previously associated with COVID-19 severity and long-COVID. Finally, the integrated model illustrates the importance of considering genetic factors in precision medicine of infectious diseases.

## Introduction

The COVID-19 pandemic had a major impact on almost all individuals and healthcare systems worldwide. As of August 2023, there have been 770,085,713 cases and nearly 6,956,173 deaths reported according to the WHO^1^. The clinical course and severity of COVID-19 disease are highly variable among individuals and includes a large spectrum of signs and symptoms, also the clinical outcomes of SARS-CoV-2 infection range from asymptomatic cases to severe respiratory failure and death^2,3^. Given the global relevance of this disease, the scientific community has aimed to identify factors influencing COVID-19 severity and long-term effects, principally focused on three main areas: viral, sociodemographic and clinical, and host-genetic factors^4–7^. Significant progress has been made in all these subjects, aiming principally to identify SARS-CoV-2 variants of concern and high-risk patient groups^8,9^. Multiple sociodemographic and clinical factors, including aging, male sex, presence of cardiovascular, respiratory, neurological, and metabolic diseases, have been associated with the clinical outcome^10^. Furthermore, multiple host-genetic factors are critical players in the COVID-19 interindividual heterogeneity^11,12^.

Early genome-wide association studies (GWAS) and case–control genetic studies identified several genomic regions, genes, and variants potentially related to COVID-19 severity^13,14^. Subsequently, numerous groups have extended, replicated and deepened such research^15,16^. In addition to identifying risk markers, these findings provide useful information to understand the pathophysiology of the disease^17,18^. Importantly, several studies have highlighted the importance of population-specific studies given different genetic backgrounds and complex genetic architectures^19,20^.

Latin-American countries were hit particularly hard by the COVID-19 pandemic. During the pre-vaccination era of SARS-CoV-2 most health systems in these countries were rapidly overwhelmed with critically ill patients and limited resources to cope with the impacts of the growing demands^21^. Colombia, among these, for example, ranked 22nd amongst 187 countries in deaths per 100,000 people by February 2022 and reported 142,780 deaths by June 2023^22,23^. Research conducted in these countries mainly focused on clinical risk profiling by assessing demographic, clinical, and virological variables^24–27^. Interestingly, machine learning approaches have also been implemented allowing to portray large-scale clinical outcomes on a nationwide scale and creating robust predictive models^28,29^. The results of these studies have been particularly useful to guide public health decisions and clinical assessment, nevertheless, COVID-19 host-genetic susceptibility factors have been relatively understudied and there are limitations in the scope of research in this regard.

Next-generation sequencing (NGS) constitutes a cost-efficient strategy to genotype a large number of variants, genes and regions simultaneously, and has been successfully applied to identify COVID-19 host-genetic risk factors in other populations^30,31^. Besides, custom NGS panels also provide a versatile tool to assess specific regions not covered by exome sequencing and to incorporate recently discovered genetic variants associated with COVID-19 outcomes. These methods have shown to be useful in identifying high-risk individuals, predicting outcomes and mortality, and they are expected to play a critical role in genomic and precision medicine^32^.

In this study, we performed a case–control analysis with the aim of characterizing clinical and host genetic factors related to disease severity and long-COVID development in a sample of the Colombian population using a custom NGS panel strategy. The results of this study suggest a positive association between multiple genetic variants and severe COVID-19 and long-term symptoms. Furthermore, we incorporated clinical and genetic factors into a predictive model useful to provide personalized risk stratification.

## Methods

### Sample selection and patients

This study enrolled 144 patients who had received a confirmed diagnosis of COVID-19 through positive reverse transcriptase polymerase chain reaction (RT-PCR), antigens, or antibodies (IgG and/or IgM specific for SARS-CoV-2) tests. Among these patients, 67 were classified as controls (non-hospitalized asymptomatic or mild COVID-19), while the remaining 77 were classified as cases due to severe or critical disease). COVID-19 clinical severity was determined in accordance with the Colombian Health Ministry guidelines^33^. Controls were selected from a private laboratory (Genética Molecular de Colombia, Bogotá D.C., Colombia) while cases were recruited amongst hospitalized patients at the Hospital Universitario Mayor-Méderi (Bogotá, Colombia). According to the literature we considered an estimated median recovery time of 21 days from COVID-19, the estimated time when viral clearance is achieved^34^. Long-COVID was defined based on the recommendation of the Nisreen Alwan Panel members, as follows: “*not recovering for several weeks or months following the start of symptoms that were suggestive of COVID*”^35^. Signs and symptoms of long-COVID were categorized using the classification developed by López-León et al.^36^.

Age is recognized as one of the main risk factors for severe COVID-19^37^. In order to minimize the impact of this variable on our findings, we restricted the age range for enrollment to individuals between 18 and 60 years old. Additionally, cases and controls were matched by age groups. Patients were invited to participate in this study between December 2020 and July 2021 and those who agreed to participate provided informed consent and underwent peripheral blood sampling. All the individuals involved in this study were not vaccinated or had received just one dose during the 7 days before the onset of symptoms.

The sample size was defined according to the minor allelic frequency (MAF) for the rs11385942 genetic variant obtained from a previous study aimed to assess three COVID-19 genetic risk variants in Colombian population^38^. The sample size was calculated using the formula *n* = Nz2*p(1-p)/α2(N-1) + z2*p(1-p) implemented in the OpenEpi web tool, using a proportion (p) of 5% (rs11385942 MAF), a confidence interval of 95% (α = 0.05, z = 1.96), and a finite population size N = 8,000,000 for the city of Bogotá^39^. The initial estimated sample size was 73 individuals, considering possible losses (e.g. loss of clinical follow-up) and the convenience of sequencing 112 samples in the available platform, this value was approximated to 144 patients to recruit. This study followed the guidelines of the Declaration of Helsinki, and all experimental procedures were approved by the Ethics Committee of Universidad del Rosario (DVO0051543-CV1334) and the technical committee of the Hospital Universitario Mayor-Méderi.

### Clinical data collection and follow-up

Demographic and clinical information was collected in standardized interviews through phone calls at least 21 days after the clinical diagnosis and test confirmation. Data included the following clinical and demographical information: age, sex, medical history, comorbidities, patient-reported symptoms, and long-term symptoms. Additionally, trained healthcare professionals performed an exhaustive revision of clinical records to confirm patients’ information and case–control classification according to the clinical guidelines. Previous pilot and training interviews were performed to minimize errors in data collection by researchers and ensure full comprehension by the participants. Biological samples from patients who completed the clinical follow-up were considered for further processing.

### DNA extraction and custom NGS panel sequencing

Genomic DNA was extracted from peripheral blood samples using the Quick-DNA™ Miniprep Plus Kit (Zymo Research) and assessed for quantity and quality. Genomic DNA was quantified using a nanodrop spectrophotometer. All samples were aliquoted and stored at 4 °C until analysis.

We performed targeted sequencing in 112 patients using a custom NGS panel. We considered two sets of target regions based on evidence reported in prospective cohorts, systematic reviews, meta-analyses, case–control analysis, GWAS and transcriptome-wide association studies (TWAS)^12,40–79^. The first set of targets were candidate genes associated with COVID-19 severity and long-term complications. The second set of targets were candidate genetic variants associated with COVID-19 severity and long-term complications. In total, 74 genes and 81 genetic variants were selected for analysis (Supplementary Table s1 and s2).

A total of 947 probes were designed using the SureDesign software, with an overall probe size of 214 bp. Hybrid capture-based enrichment of the target regions was performed using the SureSelect Custom Tier1 DNA Target Enrichment Probes (Agilent). Library preparation and capture were performed using the SureSelect XT HS2 Target Enrichment protocol (Agilent) and sequencing was performed in a DNBSEQ.G400 instrument (MGI). Enrichment, library preparation, capture and sequencing were performed by Gencell (Bogota D.C., Colombia).

### Bioinformatic analysis

The quality of the raw FASTQ files was evaluated using FastQC software (v0.10.0)^80^. Raw reads were trimmed to remove low-quality reads (< 80% Q30). Filtered reads were mapped to the reference genome GRCh37/hg19 human genome using the Burrows-Wheeler aligner (v0.17.17) and variants called using the Sentieon software package (DNAseq 202,010.02)^81,82^. The Sentieon DNAseq software is a licensed workflow used to perform variant detection implementing GATK Best Practices. The critical steps for this workflow included mapping reads to the reference genome (GRCh37/hg19), duplicates marking, indel realignment, base quality score recalibration (BQSR) and variant calling. This workflow has demonstrated strong computational performance and accuracy compared to other pipelines, including GATK^82^. The resulting Variant Call Format (VCF) files were annotated using the VarSeq software (Golden Helix)^83^. Variants were filtered according to the following quality parameters: (1) FILTER = PASS, (2) QUAL ≥ 30, and (3) Depth coverage ≥ 10X. Variants must fulfil all the previous requirements to be included in the downstream analysis. Sequencing depth and coverage were assessed using the “bedcov” function in SAMtools (v1.12)^84^.

Variant pathogenicity was classified using different approaches. First, we considered the molecular consequence of the variant categorizing as pathogenic the Loss-of-function (LoF) (frameshift, nonsense, and canonical splice site) variants. Second, for the missense variants, we used the Ensemble Method for Predicting the Pathogenicity of Rare Missense Variants (REVEL) and classified as pathogenic those with a REVEL score > 0.5^85^.

### Genetic analysis and linkage disequilibrium

We conducted two types of genetic analyses based on the set of targets. First, for the candidate variants, population genetic analyses including allelic frequencies, genotypic frequencies and Hardy–Weinberg equilibrium (HWE) were assessed using the SNPStats software^86^. The deviation of the HWE was established using a χ2 goodness-of-fit test with 1° of freedom (df). The bivariate association analysis between the candidate polymorphisms and COVID-19 severity or the presence of long-COVID was performed with the PLINK software (v1.9)^87^. The association was evaluated under several genetic models (allelic, genotypic, dominant, and recessive) using the Cochran-Armitage trend, genotypic (2df), dominant gene action (1df), and recessive gene (1df) tests. The Linkage disequilibrium (LD) between the variants localized in the same chromosome was determined by applying the D’ value in Haploview (v4.2)^88^.

Second, for candidate genes, we implemented a bioinformatic filter to identify molecular variants potentially pathogenic as mentioned previously. For these variants, populational and genetic parameters were calculated including allelic frequencies, genotypic frequencies and HWE.

### Statistical analysis and predictive model

Descriptive analysis was performed for all variables. Frequency tables were generated for qualitative variables, whereas measures of central tendency and dispersion were calculated for quantitative variables. Normality was computed by the Shapiro-Wilks test. Variables with normal distribution were expressed in terms of mean and standard deviation. Median, range and upper and lower limits were chosen if the variables did not follow normality.

A bivariate analysis was conducted to evaluate the association between clinical and host-genetics factors and COVID-19 severity and the presence of long-COVID in cases and controls. T-Student and Mann–Whitney tests were used to compare quantitative variables, whilst χ2 test was used to analyze qualitative independent variables. For genetic variants, the bivariate analysis was performed based on the following genetic models: allelic (D vs d), dominant (DD, Dd vs dd) recessive (DD vs Dd, dd), and codominant (DD vs Dd vs dd), considering (D) as the major allele and (d) as the minor allele. χ2 statistic was used with 1° of freedom for the dominant and recessive model, while 2° of freedom was selected for the genotypic model. The Cochran-Armitage test was also incorporated for genetic variables that violated HWE. Odds ratios and their respective 95% confidence interval were calculated for sociodemographic, clinical, and genetic variables.

Statistically significant variables (*p* < 0.05) selected by the bivariate analysis were chosen for the construction of the multivariate binary logistic regression model. The best model was estimated using the Stepwise Backward method^89^. Wald test was used to evaluate de significance of the individual coefficients. Model assumptions were verified, including non-collinearity, homoscedasticity, and non-error correlation. Model performance and goodness of fit were measured using the Hosmer–Lemeshow test, moreover, the discriminatory capacity of the model was tested using the ROC curve. All data processing and analysis were done using R language (v4.2), whilst PLINK was used for genetic risk modelling.

## Results

### Clinical and demographic data

**Figure 1:**
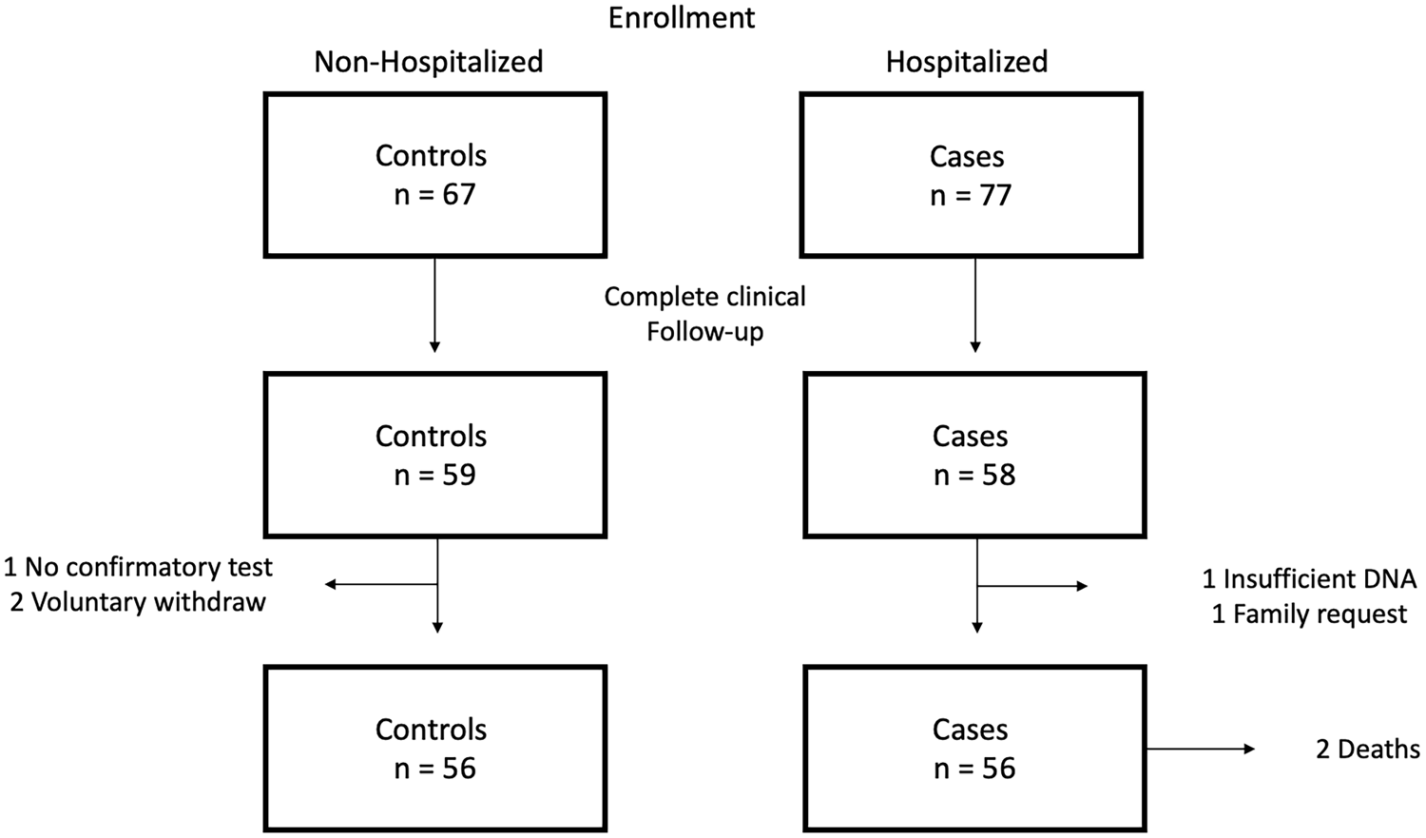
Enrollment process. The illustration depicts the process of enrollment, clinical follow-up and patient losses.

The total number of recruited patients was 144, with 77 classifieds as cases and 67 as controls. Out of these, 117 patients completed the clinical follow-up. Two patients and one family member requested voluntary withdrawal of the study, one patient had an incomplete diagnostic algorithm and another patient had insufficient DNA for analysis. Although two patients in the case group died, interviews were completed aided by family members. In the end, the analysis was performed on 56 cases and 56 controls. A summary of the enrollment process is presented in Fig. 1.

Table 1 summarizes the clinical and demographic characteristics of our study sample. The median age was similar for both cases and controls, 48 years. Men were overrepresented in the case group, accounting for 62.5% (n = 35) of cases and 42.8% (n = 24) of controls. The most frequent comorbidities were diabetes mellitus, hypertension and obesity. Additionally, 53.6% of total patients did not have any comorbidity (n = 60), whilst 19.6% (n = 22) had 2 or more. The most common symptoms in both groups were fatigue 78.6% (n = 88), musculoskeletal pain 75.9% (n = 85), headache 67.9% (n = 76), and cough 67% (n = 75). The average symptom recovery time was 23 days (± 12) for cases and 19 (± 23) for controls (Supplementary Table s3).

**Table 1:** Clinical and demographical characteristics of the studied population.

Variables	Controls (n = 56)	Cases (n = 56)	*p-*value	CI95%	OR
Age	48 (59;23)†	48 (50;21)†	0.55	–	–
Male Sex	24 (42.8%)	35 (62.5%)	0.04*	1.04–4.74	2.22
**Blood group**					
Group O	39 (69.6%)	40 (71.4%)	0.84	0.48–2.46	1.09
Group A	13 (23.2%)	14 (25.0%)	0.83	0.46–2.62	1.10
Group B	3 (5.3%)	2 (3.57%)	1	0.05–5.97	0.65
Group AB	1 (1.78%)	0 (0.0%)	1	0.00–39.00	0
**Diagnostic test**					
RT-PCR	45 (80.4%)	55 (98.2%)			
Antigen	10 (17.8%)	1 (1.8%)			
Antibodies	1 (1.8%)	0 ()			
**Comorbidities**					
BMI*	26 (34;19)†	28.4 (50;19)†	< 0.01*	0.69–4.19	–
Obesity	7 (12.5%)	17 (30.3%)	0.02*	1.15–8.09	3.05
Hypertension	8 (14.2%)	15 (26.7%)	0.1	0.85–5.70	2.20
T2DM*	3 (5.3%)	12 (21.4%)	0.02*	1.28–18.16	4.82
Cancer	3 (5.3%)	1 (1.78%)	0.62	0.03–3.19	0.32
Coronary artery disease	2 (3.57%)	1 (1.78%)	1	0.04–5.57	0.49
Arrythmia	1 (1.78%)	1 (1.78%)	1	0.06–16.39	1
Chronic kidney disease	0 (0.0%)	3 (5.3%)	0.24	–	–
HIV/Immunodeficiency	0 (0.0%)	1 (1.78%)	1	–	–
Autoimmune disease	0 (0.0%)	2 (3.57%)	0.5	–	–
Asthma	2 (3.57%)	2 (3.57%)	1	0.14–7.36	1
COPD*	0 (0.0%)	2 (3.57%)	0.5	–	–
Depression	0 (0.0%)	1 (1.78%)	1	–	–
Epilepsy/Seizures	1 (1.78%)	1 (1.78%)	1	0.06–16.39	1
Other comorbidities	24 (42.8%)	10 (17.8%)	< 0.01*	0.12–0.69	0.29
Transplanted	1 (1.78%)	2 (3.57%)	1	0.18– 23.13	2.04
Smoking history	15 (26.7%)	20 (35.7%)	0.31	0.68–3.40	1.52
Active smoker	4 (7.14%)	3 (5.3%)	1	0.16–3.45	0.74
Number of cigarettes per day	0 (40;0)†	0 (70;0)†	0.72	–	–
Number of years smoking	0 (30;0)†	0 (40;0)†	0.55	–	–
Pack year*	0 (34;0)†	0 (52;0)†	0.75	–	–
Use of medication	35 (62.5%)	31(55.3%)	0.44	0.35–1.58	0.74
Chronic steroid use	1(1.78%)	2 (3.57%)	1	0.18–23.13	2.04
Number of comorbidities	0 (4;0)†	1 (5;0)†	< 0.01*	–	–
No comorbidites	39 (69.6%)	21 (37.5%)	< 0.01*	0.12–0.57	0.26
Inverse of no comorbidities	17 (30.3%)	35 (62.5%)	< 0.01*	1.74–8.39	3.82
2 or more comorbidities	7 (12.2%)	15 (26.7%)	0.06	0.95–6.88	2.56

*Statistically significant, p-value < 0.05; COPD, Chronic obstructive pulmonary disease; BMI, body mass index; Pack year , index that measures the amount smoked over a time period; CI, confidence intervals; OR, Odds ratio; RT-PCR, Reverse transcription polymerase chain reaction.

† Variable that does not follow a normal distribution, its median was calculated (Sl Superior limit; Il Inferior limit).

Long-COVID was present in 78.5% of cases (n = 44) and 39.2% (n = 22) of controls. “Common signs and symptoms”, including fatigue, headache, insomnia, odynophagia, hair loss, weight loss and diarrhea, were the most frequent findings in both groups with 41% (n = 46), followed by “neurological signs and symptoms”, present in 33.9% (n = 38). Clinical and demographic characteristics of patients according to long-COVID status are detailed in Supplementary Table s4. The median age for long-COVID patients was 48 (21–60), whereas for patients without this sequel was 45 (23–60). The phenomenon was more frequent in females (64.1%) than in males (54.2%). The prevalence of signs and symptoms in patients with and without long-COVID is presented in Supplementary Table s5.

### Clinical association analysis

We identified multiple statistically significant associations between clinical variables and COVID-19 severity. A positive association was obtained between severe disease and male sex (*p* = 0.03; OR = 2.22; 95% CI = 1.04–4.74), obesity (*p* = 0.02; OR = 3.05; 95% CI = 1.15–8.09), type 2 diabetes mellitus (*p* = 0.02; OR = 4.82; 95% CI = 1.28–18.16) and number of comorbidities. In contrast, a negative association was observed between severe disease and no comorbidities (*p* < 0.01, OR = 0.26, 95% CI = 0.12–0.57). Specific information regarding clinical and demographic variables and their associations with COVID-19 severity is presented in Table 1.

Several symptoms showed a significant association with COVID-19 severity, including dyspnea (*p* < 0.01; OR = 16.06; 95% CI = 6.05–42.60), cough (*p* < 0.01; OR = 5.22; 95% CI = 2.16–12.65), odynophagia (*p* = 0.01; OR = 2.54; 95%; CI = 1.15–5.61), systemic symptoms, including fever (*p* < 0.01; OR = 4.64; 95% CI = 2.07–10.41) and fatigue (*p* < 0.01; OR = 7.22; 95% CI = 2.28–22.91). Time to recovery was significantly longer (*p* < 0.01) and the frequency of long-COVID signs and symptoms was higher (*p* < 0.01; OR = 5.67; 95% CI = 2.46–13.04) in the severe COVID group. Interestingly, anosmia was the only sign that showed a negative association with severe COVID-19 (*p* < 0.01; OR = 0.28; 95% CI = 0.28). Additional information about signs and symptoms according to COVID-19 severity is provided in Supplementary Table s3.

General signs and symptoms of long-COVID according to COVID-19 severity are presented in Supplementary Table s5. All variables except for anosmia/ageusia and “other signs and symptoms” were statistically significant. Among these, a strong association was found with depression (*p* < 0.01; OR = 17.15; 95% CI = 3.79–77.64), psychiatric signs and symptoms (*p* < 0.01; OR = 14.68; 95% CI = 3.24–137.49) and confusion (*p* < 0.01; OR = 13.86; 95% CI = 3.04–73.13).

The analysis of presymptomatic variables and their association with the presence of long-COVID did not yield significant results. There was no significant linkage between this outcome and different comorbidities and demographic variables, as seen in detail in Supplementary Table s4. However, dyspnea (*p* < 0.01; OR = 3.52; 95% CI = 1.59–7.77), cough (*p* = 0.01, OR = 3.12, 95% CI = 1.38–7.05), ageusia *p* = 0.03; OR = 2.39; 95% CI = 1.10–5.21) and fever (*p* < 0.01; OR = 4.94; 95% CI = 1.84–13.24) were symptoms that showed important and strong associations. Additional information can be consulted in Supplementary Table s6.

### Bioinformatic quality control

In total, we obtained 738.2 million reads, with an average of 6,591,339 reads per sample. For candidate variants, seven variants with a depth lower than 10X in more than 5% of the samples were removed (rs622568, rs1981555, rs7310667, rs11085727, rs13050728, rs113661667 and rs143334143). Genotypes of variants for patients with sequencing depth lower than 10X were designed as unknown (./.). The mean depth for the candidate variants was 996.4X (75.2X-2782.7X) (Supplementary Table s7). Regarding candidate genes, the target region spanned 179.9 Kbp and included the coding and 50 bp of flanking intronic sequence per exon. Transcript selection and variant nomenclature were based on the principal transcript identified in Ensembl^90^. Coverage above 20X was 99.05% and the average depth was 1205.9X (503.5X-1987.8X) for all the candidate genes (Supplementary Tables s8 and s9).

### Candidate variants analysis

Descriptive population genetic statistics for the candidate COVID-19 variants, including allelic and genotypic frequencies, and HWE equilibrium by case and control groups are presented in Table 2. Similarly, a descriptive analysis by presence or absence of long-COVID is reported in Supplementary Table s10. Excluding variants rs41264915, rs2232354, rs147509469, rs4424872 and rs73510898 all SNVs were found to be in HWE (93.1%; n = 67).

**Table 2:** Variant candidate analysis for COVID-19 severity. This table presents the data summary for the variants analysed in the present study.

Variant	Ref/Alt	Genomic coordinates	Closer gene	Minor allele	Allele frequency controls		Allele frequency cases		Genotype frequency controls			Genotype frequency cases			HWE	*p*-value	OR
					WT	Alt	WT	Alt	WT/WT	WT/Alt	Alt/Alt	WT/WT	WT/Alt	Alt/Alt			
rs114301457	C/T	1:155,066,988	*EFNA4*	C	1.00	0.00	0.99	0.01	0.99	0.01	0.00	0.98	0.02	0.00	1.00	0.32	NA
rs7528026	G/A	1:155,175,305	*TRIM46*	G	1.00	0.00	0.98	0.02	1.00	0.00	0.00	0.91	0.09	0.00	1.00	0.02*	NA
rs41264915	A/G	1:155,197,995	*THBS3*	A	0.97	0.03	0.98	0.02	0.96	0.02	0.02	0.98	0.0	0.02	< 0.01*	0.76	0.82
rs1123573	A/G	2:60,480,453	*BCL11A*	A	0.79	0.21	0.76	0.24	0.62	0.34	0.04	0.54	0.45	0.02	0.27	0.52	1.22
rs2232354	T/G	2:113,129,758	*IL1RN*	T	0.86	0.14	0.72	0.28	0.04	0.21	0.75	0.14	0.27	0.59	0.01*	0.01*	2.29
rs147509469	G/A	2:191,909,428	*CAVIN2, TMEFF2*	A	0.97	0.03	1.00	0.00	0.02	0.02	0.96	0.00	0.00	1.00	0.01*	0.08	0.00
rs73062389	A/G	3:45,793,925	*SLC6A20*	G	0.95	0.05	0.95	0.05	0.02	0.07	0.91	0.00	0.11	0.89	0.27	1.00	1.00
rs2271616	G/T	3:45,796,521	*SLC6A20*	T	0.87	0.13	0.89	0.11	0.75	0.23	0.02	0.82	0.14	0.04	0.19	0.54	0.77
rs2531743	G/A	3:45,796,808	*SLC6A20, LZTFL1*	A	0.71	0.29	0.77	0.23	0.46	0.48	0.05	0.55	0.43	0.02	0.89	0.29	0.72
rs72893671	T/A	3:45,809,291	*SLC6A20, LZTFL1*	T	0.96	0.04	0.91	0.09	0.91	0.09	0.00	0.82	0.18	0.00	1.00	0.18	2.09
rs17713054	G/A	3:45,818,159	*SLC6A20, LZTFL1*	G	0.99	0.01	0.92	0.08	0.98	0.02	0.00	0.84	0.16	0.00	1.00	< 0.01*	9.69
rs71325088	T/C	3:45,821,460	*SLC6A20, LZTFL1*	T	0.99	0.01	0.92	0.08	0.98	0.02	0.00	0.84	0.16	0.00	1.00	< 0.01*	9.69
rs10490770	T/C	3:45,823,240	*SLC6A20, LZTFL1*	T	0.99	0.01	0.92	0.08	0.98	0.02	0.00	0.84	0.16	0.00	1.00	< 0.01*	9.69
rs11385942	Del/A	3:45,834,968	*LZTFL1*	Del	0.99	0.01	0.91	0.09	0.98	0.02	0.00	0.82	0.18	0.00	1.00	< 0.01*	10.88
rs35081325	A/T	3:45,848,429	*LZTFL1*	A	0.99	0.01	0.92	0.08	0.98	0.02	0.00	0.84	0.16	0.00	1.00	< 0.01*	9.69
rs73064425	C/T	3:45,859,597	*LZTFL1*	C	0.99	0.01	0.92	0.08	0.98	0.02	0.00	0.84	0.16	0.00	1.00	< 0.01*	9.69
rs71325091	G/A	3:45,890,915	*LZTFL1*	G	0.96	0.04	0.92	0.08	0.93	0.07	0.00	0.84	0.16	0.00	1.00	0.15	2.35
rs13433997	T/C	3:46,008,273	*FYCO1, XCR1*	T	0.92	0.08	0.88	0.12	0.84	0.16	0.00	0.77	0.23	0.00	0.60	0.27	1.67
rs34438204	T/C	3:46,039,814	*XCR1*	T	0.96	0.04	0.92	0.08	0.91	0.09	0.00	0.84	0.16	0.00	1.00	0.27	1.87
rs7642320	A/G	3:46,049,130	*XCR1*	A	0.88	0.12	0.86	0.14	0.77	0.23	0.00	0.71	0.29	0.00	0.21	0.55	1.26
rs9877748	A/G	3:46,069,589	*XCR1*	A	0.91	0.09	0.88	0.12	0.84	0.14	0.02	0.77	0.23	0.00	1.00	0.51	1.33
rs13069742	A/G	3:46,072,724	*XCR1*	A	0.91	0.09	0.88	0.12	0.84	0.14	0.02	0.77	0.23	0.00	1.00	0.51	1.33
rs35110864	G/A	3:46,112,965	*XCR1, CCR1*	G	0.96	0.04	0.92	0.08	0.91	0.09	0.00	0.84	0.16	0.00	1.00	0.27	1.87
rs13085367	T/C	3:46,131,332	*XCR1, CCR1*	T	0.96	0.04	0.92	0.08	0.093	0.07	0.00	0.86	0.12	0.02	0.31	0.15	2.35
rs4443214	T/C	3:46,136,372	*XCR1, CCR1*	T	0.94	0.06	0.91	0.09	0.88	0.12	0.00	0.84	0.14	0.03	0.48	0.62	1.27
rs35775079	C/T	3:46,220,620	*CCR3*	C	0.99	0.01	0.96	0.04	0.98	0.02	0.00	0.91	0.09	0.00	1.00	0.02*	8.53
rs11919389	T/C	3:101,705,614	*RPL24*	T	0.79	0.21	0.81	0.19	0.61	0.36	0.04	0.66	0.30	0.04	1.00	0.52	0.80
rs343320	G/A	3:146,517,122	*PLSCR1*	G	0.91	0.09	0.96	0.04	0.84	0.15	0.02	0.93	0.07	0.00	0.35	0.09	0.37
rs56162149	C/T	5:131,995,059	*ACSL6*	C	0.83	0.17	0.79	0.21	0.68	0.30	0.02	0.64	0.29	0.07	0.55	0.40	1.33
rs9271609	T/C	6:32,623,820	*HLA-DRB1*	T	0.66	0.34	0.65	0.35	0.36	0.61	0.04	0.46	0.38	0.16	0.41	0.78	1.08
rs2496644	A/C	6:41,515,007	*LINC01276*	C	0.74	0.26	0.73	0.27	0.54	0.41	0.05	0.54	0.39	0.07	0.81	0.88	1.04
rs1886814	A/C	6:41,534,945	*FOXP4*	A	0.77	0.23	0.77	0.23	0.59	0.36	0.05	0.59	0.36	0.05	1.00	1.00	1.00
rs28368148	C/G	9:21,206,606	*IFNA10*	C	1.00	0.00	0.99	0.01	1.00	0.00	0.00	0.98	0.02	0.00	1.00	1.00	1.00
rs505922	T/C	9:133,273,813	*ABO*	T	0.84	0.16	0.82	0.18	0.70	0.29	0.02	0.68	0.29	0.04	1.00	0.72	1.13
rs529565	T/C	9:133,274,084	*ABO*	T	0.84	0.16	0.78	0.22	0.70	0.29	0.02	0.61	0.34	0.05	1.00	0.24	1.50
rs61882275	G/A	11:34,482,745	*ELF5*	G	0.61	0.39	0.54	0.46	0.32	0.57	0.11	0.32	0.45	0.23	0.70	0.34	1.29
rs10774671	G/A	12:112,919,388	*OAS1*	A	0.76	0.24	0.82	0.18	0.57	0.38	0.05	0.71	0.21	0.07	0.25	0.25	0.68
rs2660	G/A	12:112,919,637	*OAS1*	A	0.82	0.18	0.85	0.15	0.66	0.32	0.20	0.77	0.16	0.07	0.18	0.59	0.82
rs10850097	C/T	12:112,923,312	*OAS1*	T	0.79	0.21	0.84	0.16	0.61	0.36	0.04	0.75	0.18	0.07	0.21	0.40	0.74
rs6489867	C/T	12:112,925,745	*OAS1*	T	0.77	0.23	0.82	0.18	0.57	0.39	0.04	0.73	0.18	0.09	0.24	0.32	0.71
rs7955267	C/T	12:112,941,234	*OAS3*	T	0.79	0.21	0.80	0.20	0.59	0.39	0.02	0.71	0.18	0.11	0.24	0.74	0.89
rs56106917	C/Del	12:132,489,231	*FBRSL*	C	0.74	0.26	0.71	0.29	0.57	0.34	0.09	0.50	0.41	0.09	0.48	0.55	1.19
rs9577175	C/T	13:112,889,041	*ATP11A*	C	0.79	0.21	0.72	0.28	0.59	0.39	0.02	0.50	0.45	0.05	0.21	0.28	1.40
rs4424872	T/A	15:93,046,840	*RGMA*	A	0.96	0.04	0.97	0.03	0.95	0.02	0.04	0.96	0.02	0.02	< 001*	0.47	0.58
rs117169628	G/A	16:89,196,249	*SLC22A31*	G	0.88	0.12	0.87	0.13	0.77	0.23	0.00	0.79	0.16	0.05	0.47	0.69	1.17
rs79600142	T/C	17:45,820,356	*CRHR1*	T	0.87	0.13	0.94	0.05	0.73	0.27	0.00	0.89	0.09	0.02	1.00	0.07	0.43
rs62054835	A/C	17:45,857,306	*MAPT-AS1*	A	0.87	0.13	0.95	0.05	0.73	0.27	0.00	0.91	0.07	0.02	1.00	0.04*	0.36
rs112572874	A/G	17:45,995,618	*MAPT*	A	0.86	0.14	0.94	0.06	0.71	0.29	0.00	0.89	0.09	0.02	1.00	0.04*	0.40
rs1819040	T/A	17:46,142,465	*KANSL1*	T	0.85	0.15	0.94	0.06	0.73	0.23	0.04	0.89	0.09	0.02	0.11	0.03*	0.37
rs2532300	T/C	17:46,152,620	*KANSL1*	T	0.87	0.13	0.94	0.06	0.73	0.27	0.00	0.89	0.09	0.02	1.00	0.07	0.43
rs3848456	C/A	17:49,863,260	*TAC4*	C	0.75	0.25	0.77	0.23	0.52	0.46	0.02	0.57	0.39	0.04	0.12	0.75	0.90
rs77534576	C/T	17:49,863,303	*TAC4*	C	0.83	0.17	0.85	0.15	0.66	0.34	0.00	0.73	0.23	0.04	0.73	0.72	0.87
rs12610495	A/G	19:4,717,660	*DPP9*	A	0.78	0.22	0.71	0.29	0.61	0.34	0.05	0.55	0.32	0.12	0.21	0.28	1.39
rs2109069	G/A	19:4,719,431	*DPP9*	G	0.75	0.25	0.70	0.30	0.55	0.39	0.05	0.52	0.36	0.12	0.48	0.37	1.30
rs2277732	C/A	19:4,723,658	*DPP9*	C	0.77	0.23	0.71	0.29	0.59	0.36	0.05	0.54	0.34	0.12	0.33	0.29	1.38
rs4804803	A/G	19:7,747,847	*CD209*	A	0.82	0.18	0.85	0.15	0.68	0.29	0.04	0.75	0.20	0.05	0.18	0.59	0.82
rs73510898	G/A	19:10,305,768	*ZGLP1*	G	0.96	0.04	0.96	0.04	0.91	0.09	0.00	0.95	0.02	0.04	0.01*	1.00	1.00
rs74956615	T/A	19:10,317,045	*RAVER1*	T	0.94	0.06	0.98	0.02	0.88	0.12	0.00	0.96	0.04	0.00	1.00	0.09	0.27
rs34536443	G/C	19:10,352,442	*TYK2*	G	0.96	0.04	0.98	0.02	0.93	0.07	0.00	0.96	0.04	0.00	1.00	0.41	0.49
rs429358	T/C	19:44,908,684	*APOE*	T	0.88	0.12	0.95	0.05	0.79	0.18	0.04	0.89	0.11	0.00	0.20	0.11	0.46
rs368565	C/T	19:48,697,960	*FUT2*	T	0.66	0.34	0.51	0.49	0.48	0.36	0.16	0.36	0.29	0.35	0.04*	0.02*	1.87
rs4801778	G/T	19:48,867,352	*PLEKHA4*	G	0.85	0.15	0.88	0.12	0.73	0.23	0.04	0.77	0.21	0.02	0.44	0.56	0.79
rs17860115	C/A	21:33,230,000	*IFNAR2*	C	0.52	0.48	0.48	0.52	0.25	0.54	0.21	0.25	0.46	0.29	1.00	0.59	1.15
rs2300370	G/A	21:33,232,252	*IFNAR2*	G	0.54	0.46	0.46	0.54	0.27	0.54	0.20	0.23	0.46	0.30	1.00	0.29	1.33
rs2252639	A/G	21:33,245,424	*IFNAR2*	A	0.52	0.48	0.53	0.47	0.23	0.57	0.20	0.29	0.48	0.23	0.70	0.89	0.96
rs2236757	A/G	21:33,252,612	*IFNAR2*	G	0.54	0.46	0.52	0.48	0.25	0.57	0.18	0.30	0.43	0.27	0.18	0.79	1.07
rs2300371	C/T	21:33,259,936	*IFNAR2*	C	0.54	0.46	0.55	0.45	0.25	0.57	0.18	0.34	0.43	0.23	1.00	0.79	0.93
rs8178521	C/T	21:33,287,378	*IL10RB*	C	0.76	0.24	0.74	0.26	0.57	0.38	0.05	0.61	0.27	0.12	0.13	0.64	1.15
rs35370143	Del/Ins	21:33,959,663	*LINC00649*	Del	0.83	0.17	0.79	0.21	0.66	0.34	0.00	0.62	0.34	0.02	0.35	0.49	1.26
rs2298660	C/T	21:41,473,706	*TMPRSS2*	C	0.79	0.21	0.81	0.19	0.62	0.34	0.04	0.68	0.27	0.05	0.76	0.74	0.89
rs2298661	C/A	21:41,473,715	*TMPRSS2*	C	0.78	0.22	0.82	0.18	0.61	0.34	0.05	0.70	0.25	0.05	0.38	0.40	0.75
rs3787946	G/C	21:41,475,808	*TMPRSS2*	C	0.78	0.22	0.80	0.20	0.61	0.34	0.05	0.66	0.29	0.05	0.57	0.62	0.85

Alt, Alternative allele; HWE, Hardy–Weinberg equilibrium; OR, Odds ratio; Ref/Alt, Reference/Alternative, WT, Wild-type allele.

The association analysis under the allelic genetic model between candidate variants and COVID-19 severity revealed that 13 variants were significantly associated with the worst outcome (rs7528026, rs2232354, rs17713054, rs71325088, rs10490770, rs11385942, rs35081325, rs73064425, rs35775079, rs62054835, rs112572874, rs1819040, rs368565). The variants with the strongest association strength were rs11385942 (*p* < 0.01; OR = 10.88; 95% CI = 1.36–86.51), rs10490770 (*p* < 0.01; OR = 9.69; 95% CI = 1.20–77.89), rs35081325 (*p* < 0.01; OR = 9.69; 95% CI = 1.20–77.89), rs71325088 (*p* < 0.01; OR = 9.69; 95% CI = 1.20–77.89) and rs73064425 (*p* < 0.01; OR = 9.69; 95% CI = 1.20–77.89) located in or close to *LZTFL1,* and rs35775079 (*p* = 0.02; OR = 8.53; 95% CI = 1.05–69.45) located in *CCR3* (Table 2). Due to the close genomic proximity of several candidate variants, we assessed linkage disequilibrium (LD) strength with r 2 and Lewontin's D' statistic using the Haploview software. For variants located in chromosome 3, five of them (rs17713054, rs71325088, rs10490770, rs35081325, rs73064425) displayed LD with D' values of 1 or close to 1 (Supplementary Figure s1A). Likewise, three of the associated variants in chromosome 17 were found to be in linkage disequilibrium (rs62054835, rs112572874, rs1819040) (Supplementary Figure s1B).

The association analysis under the allelic genetic model between candidate variants and long-COVID identified 4 variants associated with this clinical condition (rs147509469, rs9577175, rs368565, rs8178521). The variants with the strongest association strength were rs8178521 located in *IL10RB* (*p* = 0.01; OR = 2.51; 95% CI = 1.27–4.94) and rs9577175 (*p* = 0.04; OR = 1.99; 95% CI = 1.034–3.83) located in the genomic region 13:112,889,041 (Supplementary Table s10). These associated variants were located in different chromosomes therefore no LD analyses were conducted.

### Candidate genes analysis

A total of 291 variants were identified in the 74 candidate genes related to severe COVID-19 or long-COVID. After our filtering strategy, we obtained 65 variants, from which 69.2% (n = 45) correspond to LoF variants and 30.8% (n = 20) stand for predicted pathogenic missense variants (REVEL score > 0.5). Regarding LoF variants, *TLR3* was the gene harboring the higher number of variants (n = 9) followed by *MUC1* (n = 7). All the other genes had less than 5 LoF variants. Likewise, *FOXP4* was the gene accounting for the highest number of predicted pathogenic missense variants (n = 3) followed by *DPP4 and FUT2* with 2 variants each (Table 3, Supplementary Table s11). LoF variant frequencies among the cases were slightly higher (n = 36) than in the controls (n = 30), nevertheless, this difference was not statistically significant (*p* = 0.33). Conversely, the number of predicted missense pathogenic variants in the control group was higher than in the group of cases (n = 34 vs n = 22), with a significant difference (*p* = 0.03). On the other hand, several genes such as *TLR3* (n = 6), *OAS3* (n = 2) and *APOE* (n = 1) presented LoF variants exclusively in the case group. Similarly, *THBS3* (n = 1) and *ATP11A* (n = 1) harbored predicted missense pathogenic variants exclusively within the case group.

**Table 3:** Number of patients with potential pathogenic variants per gene according to COVID-19 severity.

Gen	LoF variants		Predicted pathogenic missense variants		Total
	Cases	Controls	Cases	Controls	
*APOE*	0	0	0	0	0
*APOL1*	0	0	0	0	0
*ARHGAP27*	0	0	0	0	0
*ARL17B*	1	1	0	0	2
*ATP11A*	1	1	1	0	3
*BCL11A*	0	0	0	0	0
*CCR3*	0	0	0	0	0
*CCR5*	0	0	0	0	0
*CCR9*	0	0	0	0	0
*CD14*	0	0	0	1	1
*CENPS*	0	0	0	0	0
*CFAP73*	0	0	0	0	0
*DPP4*	2	2	1	1	6
*DPP9*	0	0	0	0	0
*FCGR2A*	1	2	0	0	3
*FDX2*	0	0	0	0	0
*FOXP4*	1	0	2	2	5
*FURIN*	0	0	0	0	0
*FUT2*	1	0	13	17	31
*FYCO1*	0	0	0	0	0
*HSD17B14*	1	0	0	1	2
*ICAM1*	0	0	0	0	0
*ICAM3*	0	0	0	0	0
*ICAM5*	0	1	0	0	1
*IFITM3*	0	0	0	0	0
*IFNA10*	0	0	0	0	0
*IFNAR1*	0	0	0	0	0
*IFNAR2*	0	2	0	0	2
*IRF3*	0	0	0	0	0
*IRF7*	0	0	0	0	0
*KANSL1*	0	0	0	0	0
*KLRC2*	0	0	0	0	0
*LZTFL1*	1	0	0	0	1
*MAPT*	0	0	0	0	0
*MUC1*	5	6	0	0	11
*MX1*	1	0	0	0	1
*NAPSA*	0	0	0	1	1
*NCOA4*	0	0	0	0	0
*NUCB1*	0	0	0	0	0
*OAS1*	0	0	0	0	0
*OAS3*	2	0	0	2	4
*OCLN*	0	2	0	0	2
*PDE4A*	0	0	0	1	1
*PIGN*	1	0	0	1	2
*PLSCR1*	1	1	0	0	2
*PPP1R15A*	0	0	0	0	0
*RPL24*	0	0	1	0	1
*SLC30A5*	1	0	1	0	2
*SLC6A20*	0	0	0	0	0
*SPDEF*	0	0	0	0	0
*TAC4*	5	4	0	0	9
*TBK1*	1	0	0	0	1
*THBS3*	0	0	1	0	1
*TICAM1*	0	0	0	0	0
*TLR3*	6	0	0	1	7
*TLR7*	0	0	0	0	0
*TMEM65*	1	0	0	0	1
*TMPRSS2*	0	0	0	0	0
*TRIM46*	0	0	0	0	0
*TYK2*	0	0	2	5	7
*UGT2A1*	1	4	0	0	5
*UGT2A2*	0	0	0	1	1
*UNC93B1*	1	4	0	0	5
*WNT3*	0	0	0	0	0
*XCR1*	0	0	0	0	0
*ZNF561*	0	0	0	0	0
Total	35	30	22	34	121

Concerning the assessment of potential deleterious variant frequencies between patients with and without long-COVID, there were no significative differences in LoF (*p* = 0.27) or predicted pathogenic missense variants (*p* = 0.70) frequencies. Nevertheless, some genes like *UGT2A1* (n = 5), *PLSCR1*, and *ARL17B* (n = 2) showed LoF variants exclusively in the long-COVID group. Likewise, *FOXP4* (n = 4), and *TLR3* (n = 1) harbored predicted pathogenic missense variants only in the long-COVID group (Supplementary Table s12).

Extended information about potential pathogenic variants in candidate genes is presented in Supplementary Table s13. Notably, we identified a novel variant (NM_030930.4: c.1360 + 2 T > A) in *UNC93B1* exclusively present in patients from the control group with an allelic frequency of 8.93% and in patients belonging to the non long-COVID group with a frequency of 8.70%. This variant showed a significant association with asymptomatic/mild COVID-19 (*p* = 0.02) and no long-COVID clinical outcome (*p* = 0.01) and has been previously associated with influenza susceptibility^61,91^.

### Predictive models

**Figure 2:**
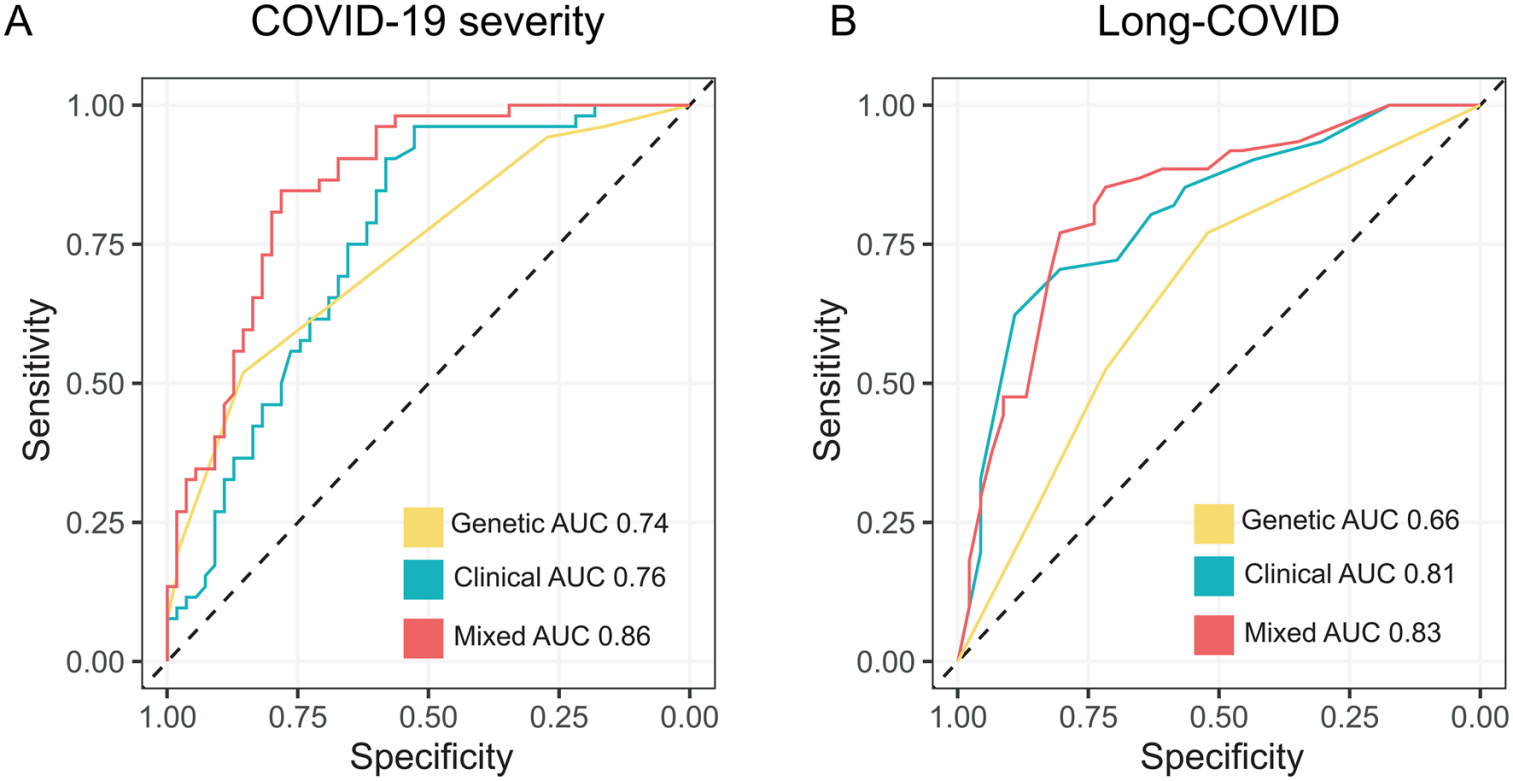
Predictive model ROC curves. Comparison of receiver operating characteristic curve (ROC) curves derived from the different predictive models. ROC curves for clinical, genetic, and mixed predictive models for COVID-19 severity (**A**) and long-COVID (**B**).

Genetic and clinical variables with significant association with the outcomes of interest, severe COVID-19 and long-COVID, were incorporated into binary logistic regression models. Three different predictive models for each of our main outcomes were built, a clinical model, a genetic model, and a mixed model. The best model was selected according to the Akaike information criteria (AIC) using the stepwise backward method. These comparisons showed that the mixed models have the best discriminatory power, both for severity (AUC = 0.86; 95% CI = 0.78–0.93) and for long COVID (AUC 0.83; 95% CI = 0.74–0.91). A complete comparison of these models, including selected variables, is shown in Tables 4, 5, and Fig. 2. Quality and model assumptions were validated identifying the absence of collinearity, with the variance inflation factor test (< 1.2), homoscedasticity, with the Breusch-Pagan test (*p* > 0.05), calibration with the Hosmer–Lemeshow test (*p* > 0.05), and error independence, with the Durbin-Watson test (*p* > 0.05).

**Table 4:** Comparison of clinical, genetic and mixed models for COVID-19 severity.

Predictors	Clinical model					Genetic model					Mixed model				
	OR	SD	95% CI	Statistic	p-value	OR	SD	95% CI	Statistic	p-value	OR	SD	95% CI	Statistic	p-value
Intercepts	0.02	0.03	0.00–0.47	–2.40	0.01	7.74	9.29	0.74–81.39	1.70	0.08	0.13	0.30	0.00–11.83	–0.89	0.37
Male sex	2.62	1.16	1.10–6.24	2.18	0.02						2.72	1.36	1.02–7.27	1.99	0.04
BMI	1.16	0.07	1.03–1.29	2.55	0.01						1.17	0.08	1.02–1.33	2.29	0.02
No comorbidity	0.49	0.23	0.20–123	–1.52	0.12						0.37	0.20	0.13–1.05	–1.87	0.06
rs2232354						0.35	0.17	0.14–0.89	–2.22	0.42	0.42	0.22	0.15–1.18	–1.64	0.10
rs1819040						4.71	2.97	1.37–16.21	0.01	5.87	5.87	4.11	1.49–23.15	2.53	0.01
rs11385942						0.06	0.06	0.01–0.52	0.01	0.03	0.03	0.04	0.00–0.37	–2.78	0.00
**Comparison**															
Observations	107					107					107				
Deviation	126.44					126.82					104.43				
AIC	134.44					134.82					118.43				
Log Likelihood ratio	–63.22					–63.41					–52.21				

AIC, Akaike information criteria; CI, confidence interval; OR, Odds Ratio; SD, Standard deviation.

**Table 5:** Comparison of clinical, genetic and mixed models for long-COVID.

Predictors	Clinical model					Genetic model					Mixed model				
	OR	SD	95% CI	Statistic	p-value	OR	SD	95% CI	Statistic	p-value	OR	SD	95% CI	Statistic	p-value
Intercept	0.08	0.06	0.02–0.31	–3.68	< 0.01	3.36	1.34	1.54–7.34	3.04	< 0.01	0.18	0.14	0.04–0.79	–2.27	0.02
Anosmia	4.12	2.06	1.55–11	2.83	< 0.01						3.23	1.67	1.17–8.89	2.27	0.02
Fever > 38 °C	2.04	0.99	0.79–5.29	1.47	0.14						2.25	1.13	0.84–6.02	1.61	0.10
Fatigue	3.82	2.28	1.19–12.28	2.25	0.02						3.21	1.98	0.96–10.75	1.89	0.05
Clinical severity	4.42	2.24	1.64–11.92	2.94	0.00						4.98	2.63	1.77–14.02	3.04	< 0.01
rs9577175						0.54	0.22	0.24–1.20	–1.51	0.13					
rs8178521						0.38	0.16	0.17–0.87	–2.28	0.02	0.35	0.18	0.13–0.95	–2.06	0.04
**Comparison**															
Observations	107					107					107				
Deviation	113.22					137.48					108.79				
AIC	123.22					143.48					120.79				
Log Likelihood ratio	− 56.61					− 68.74					− 54.39				

Note: AIC, Akaike information criteria; CI, confidence interval; OR, Odds Ratio; SD, Standard deviation.

For the severity COVID-19 predictive mixed model the variables included were sex, body mass index (BMI), presence of comorbidities, and the genetic variants rs2232354, rs11385942 and rs1819040, belonging to the genes *IL1RN*, *LZTFL1* and *KANSL1*, respectively. The resulting predicting score is presented in Eq. 1.

1 $$Adjusted \,score=\frac{1}{1+{e}^{\begin{array}{c}-(-2.04+0.99\left(male\right)+0.15\left(BMI\right)+1\left(comorb\right)+\\ 3.36(rs11385942 WT/Alt)+0.86(rs2232354 WT/Alt)-1.77(rs1819040 WT/Alt))\\ \end{array}}}$$

COVID-19 severity predictive model. Where the adjusted score is a number between 0 and 1, “*male*” male sex, “*BMI*” body mass index, “*comorb*” presence of comorbidities and “*WT/Alt*” the presence of the alternative allele for each genetic variant.

For the long-COVID predictive mixed model, the variables included were anosmia, fever, fatigue, COVID-19 clinical severity and presence of rs8178521 in the *IL10RB* gene. The resulting predicting score is presented in Eq. 2.

$$Adjusted \,score=\frac{1}{1+{e}^{\begin{array}{c}-(-1.72+1.6\left(severe COVID-19\right)+1.17\left(anosmia\right)+1.16\left(fatigue\right)+0.8\left(fever\right)+\\ 1.06(rs8178521 WT/Alt))\\ \end{array}}} (2)$$

COVID-19 predictive model. Where the adjusted score is a number between 0 and 1, “severe COVID-19” presence of severe disease, “anosmia”, “fatigue” and “fever” refer to the presence of these symptoms and “*WT/Alt*” presence of the rs8178521 variant.

## Discussion

In just a matter of months, SARS-CoV-2 emerged as one of the most critical public health emergencies of the twenty-first century. Despite substantial progress in the understanding of this disease, the significant phenotypic variation in host responses and outcomes has not been fully elucidated^2,3,29^. This variability is influenced by several factors, encompassing viral and host-related characteristics. Host genetic factors constitute important risk factors for COVID-19 severity, mortality, and the presence of sequels. It is important to note that these genetic factors have remained understudied in Latin-American countries. In this study, we aim to characterize clinical and host genetic factors related to disease severity and long-COVID development in a sample of the Colombian population. We identified multiple genetic and non-genetic risk factors associated with these outcomes. Furthermore, we incorporated these factors into two predictive models for our outcomes: disease severity and long-COVID. This study illustrates the potential usefulness of a combined strategy using clinical and genomic data to identify high-risk individuals in a specific population.

Several non-genetic factors have demonstrated a substantial association with severe COVID-19 disease, including male sex, advanced age, and the presence of various comorbidities^92^. Our study supports such findings, revealing a significant association between male sex, obesity, and diabetes mellitus with more adverse outcomes. There is growing evidence suggesting that comorbidities play a role in the development of endothelial damage, promoting a prothrombotic and inflammatory status and higher viral replication, ultimately exacerbating clinical outcomes^93,94^. Regarding the clinical manifestations of the disease, respiratory and systemic signs and symptoms, including dyspnea, cough, odynophagia, fever, and fatigue, have shown a significant association with severe COVID-19 cases^95^. This association can be attributed to the immune-cytopathic effect of the virus on lung tissue. This effect leads to a systemic proinflammatory response and widespread viral dissemination, which, in turn, exacerbates symptoms through multiorgan involvement^96^. Interestingly, our study identified anosmia as a protective factor for severe disease, an observation previously made in other studies^97^.

Regarding non-genetic factors and long-COVID, we found that severe COVID-19 is associated with a higher prevalence of long-COVID, as previously reported^98^. We did not find any additional statistically significant associations between long COVID and either presymptomatic clinical or demographic variables, contrary to what has been reported in other studies. Previous research has indicated that patients over 50 years old and those with multiple comorbidities are more likely to experience long-COVID^99^. We believe that these discrepancies are due to differences in the methodological design, as these conclusions have been mostly based on considerably older patients^100,101^. On the other hand, clinical manifestations during the acute phase of the disease, including respiratory and systemic signs and symptoms, show a correlation with long-COVID, in agreement with previous reports^102^. This finding suggests that these acute-phase symptoms might serve as indicators of vascular, pulmonary, and central nervous system damage^99^.

To date, it has been recognized that the response to COVID-19 infection is influenced by host genetic factors. Evidence from a study of twins, for instance, suggests a 50% heritability of COVID-19 risk^103^. Given the implication of these factors, several initiatives have been developed to identify risk variants and genes associated with COVID-19 severity and mortality. The methods include GWAS, whole exome sequencing, whole genome sequencing, and case–control associations^14,43^. Importantly, several authors have highlighted the limitations of these studies concerning the small number of variants or genes assessed and the underrepresentation of Latin-American populations. To the best of our knowledge, our study is the first to incorporate a custom NGS technique to evaluate host genetic factors contributing to both COVID-19 severity and long COVID within a Latin-American sample.

This study identified 13 genetic variants associated with COVID-19 severity. Several of these variants, mainly located in the critical *loci* 3p21.31 and 17q21.31, have been described as important risk factors. In agreement with previous GWAS studies, we found that rs11385942, an intron variant located in *LZTFL1*, shows the strongest association (*p* < 0.01; OR = 10.88) with severe or critical COVID-19^43,63,104^. These findings support the utility of this risk allele as a useful molecular prognostic biomarker in diverse populations. Conversely, we identified rs1819040, a variant located in *KANSL1*, as a protective allele against severe or critical disease (*p* = 0.03; OR = 0.37), as previously reported in other studies^43^. This variant was found in linkage disequilibrium with two intronic variants, rs62054835, and rs112572874, located in *MAPT-AS1* and *MAPT*, respectively. Transcriptome-wide association studies, GWAS, and eQTL studies have suggested the role of *MAPT* as a susceptibility gene for severe COVID-19^48^. Indeed, genetic variants within *MAPT* have been related to autoimmune diseases, normal lung function, and interstitial lung disease^105,106^. Additionally, we found a significant association between severe COVID-19 and rs35775079, a variant located in the intronic region of *CCR3* (*p* = 0.02; OR = 8.53). *CCR3* encodes a chemokine receptor highly expressed in eosinophils, basophils, TH1 and TH2 CD4 + T cells, and airway epithelial cells^107^. This receptor is an important mediator of allergic responses and genetic mouse model studies have demonstrated its crucial role in airway inflammatory cell infiltration^107,108^. It has been proposed that variants in this gene may impact the disease outcome through an excessive inflammatory response, one of the hallmarks of severe COVID-19, as well as of other severe respiratory virus infections^109,110^.

Currently, long-COVID symptoms are recognized as common sequelae of COVID-19 and represent a crucial focus of ongoing research. Similar to other reports, our research identifies an overall incidence of long-COVID, approximately 80% among non-severe COVID-19 patients and 40% among those with severe cases^111^. Remarkably, we identified 4 genetic variants associated with this clinical condition. The variant with the strongest association, rs8178521, is located within the *IL10RB* gene (*p* = 0.01; OR = 2.51). This variant has been previously linked to COVID-19 severity^45^. However, our study represents the first report suggesting its potential association with long-COVID. *IL10RB* encodes for a receptor of type III interferons and plays a pivotal role in immunomodulation through its regulation of IL-10 influencing the differentiation, proliferation, and cytokines production of mast cells^112^. Moreover, recent reports have suggested that the deregulated release of inflammatory mediators by mast cells is one of the potential mechanisms underlying the development of long-COVID^113,114^.

Our study identified 70 potential deleterious rare variants in candidate genes associated with the pathogenesis and immune response against SARS-CoV-2 infection. Rare and low-frequency variants have been shown to contribute to COVID-19 and other immune-related complex disorders^115,116^. However, despite these associations, our study did not find any significant difference in variant frequency within our study sample. This lack of significance could be attributed to limitations in the sample size of patients included in our study. Intriguingly, some of the LoF variants identified in this study were exclusively present in patients with severe or critical COVID-19. *TLR3*, for example, harbored 9 LoF variants in the case group compared to 0 among the controls. Other genetic variants in *TLR3*, such as rs3775291, have been related to an impairment in the immune response and associated with COVID-19 susceptibility and mortality^117^. Given the protective role of *TLR3* and its function in innate immunity during SARS-CoV-2 infection, other potentially deleterious variants could similarly influence COVID-19 clinical outcomes. Likewise, we identified a potential deleterious missense variant, *UGT2A1* c.576 T > A, (rs111696697) exclusively in patients with long-COVID (allele frequency 0.75). This gene is expressed in the olfactory epithelium and codifies for a protein member of the UDP-glycosyltransferase family which plays an important role as an odorant metabolizing enzyme^118^. Furthermore, *UGT2A1*/U*GT2A2* has been associated with COVID-19 anosmia, one of the most frequent long COVID symptoms^70^. It should be highlighted that although some clinical and paraclinical predictors of long-COVID have been identified, the genetic factors related to this condition remain largely unknown. Identifying such factors could be useful to illuminate the biological and molecular basis of this disease.

In addition to genetic host variants, numerous studies have highlighted the role of viral genetic factors in COVID-19 pathogenicity, infectivity, and outcomes^119,120^. The appearance of variants of concern (VOC) and variants of interest (VOI), in particular, has been continuously monitored and evaluated since the beginning of the epidemic^121,122^. Although our study did not examine viral genetic factors, genomic surveillance studies conducted during the collection period of the samples (December 2020—July 2021) in Bogotá, indicated that the predominant variants were B.1.621 (Mu) 57.3% (469/819), P.1 (Gamma) 14% (114/819), and B.1.1.7 (alpha) 2.8% (23/819)^22^. Therefore, the variant most detected during this period was Mu. This variant was later classified as a variant being monitored (VBM) by the Centers for Disease Control and Prevention (CDC U.S.) and had no reports of significant effects of this variant on infectivity, transmissibility, or severity in contrast to VOIs. While complex viral and host genetic interactions cannot be discarded, we estimate that patients among the groups can be compared since they were enrolled during the same period, when the previously mentioned viral variants were circulating. On the other hand, although Bogota is a large city, with an area of 1636 km^2^, the Hospital Universitario Mayor-Mederi and the private laboratory Genética Molecular de Colombia, where cases and controls were enrolled, respectively, are just 8 km away from each other. Also, it should be highlighted that controls were recruited from a different location than cases, given that Colombian healthcare policies advised to not attend hospitals for mild COVID-19 symptoms. As a result, there were limited options to include mild cases from hospital settings.

As depicted, the hybrid models combining both clinical and genetic host variables constitute strong and reliable tools to predict COVID-19 outcomes. The biological basis of clinical variables has been discussed in previous models and reviews^123–125^. On the other hand, recent studies have integrated specific genetic variants into predictive models^38,126^. It is to be noted that the inclusion of variants from the *IL1RN* and *KANSL1* genes in our model represents a novel approach. The absence of these variants in previous models may reflect differences in the genetic background of the studied populations and the complexity of the genetic architecture underlying COVID-19 outcomes. Thus, this study suggests that such a multivariable approach is a useful and innovative tool to identify high-risk individuals and prioritize limited health resources. We believe that such approaches are consistent with genomic and personalized medicine initiatives and may be useful for future pandemics.

## Conclusions

This study analyzed the association between genetic and non-genetic factors with COVID-19 severity and the presence of long-COVID in a sample of the Colombian population. We found an association between these two outcomes and several genetic and non-genetic factors. The risk genetic variants are located in genes whose products participate in immunological signaling and humoral response against microorganisms. We highlight the usefulness of combining clinical and genomics data to develop models to predict COVID-19 response. Applying these predictive models in the clinical setting can help to identify high-risk individuals and focus resources and actions to reduce morbidity and mortality.

### Limitations

Among the limitations of this study, we should mention that although the sample size might be sufficient to identify genetic variants with a medium or large effect, it may have been underpowered to detect the association of low-effect variants. The sample size, also, was calculated based on the available information on allele frequency. Third, we noticed that after the custom panel was designed and the probes were synthesized, novel candidate variants and genes were described in the literature. These were not included in this study and this fact highlights the importance of periodically updating NGS custom panel with clinical applications. On the other hand, although we took several measures to reduce potential bias, this may have been introduced during the interviews or clinical data collection. Finally, we should underline that the proposed models were not validated in a larger cohort, thus, more studies will be necessary to evaluate their accuracy and precision.

### Supplementary Information

Supplementary Information 1.

Supplementary Information 2.

## Data Availability

The datasets generated and/or analysed during the current study are not readily available because the nature of this research contains information that could compromise the participants’ privacy and they did not agree to share their data publicly. Requests to access the datasets should be directed to Oscar Ortega-Recalde (oortegar@unal.edu.co).
