# Supplementary material for: Next-generation sequencing of host genetics risk factors associated with COVID-19 severity and long-COVID in Colombian population
Source: Sci Rep. 2024 Apr 11;14:8497. doi: 10.1038/s41598-024-57982-3 (PMC11009356; doi:10.1038/s41598-024-57982-3)
Supplement: Supplementary file 1 — Supplementary Information 1. [file 41598_2024_57982_MOESM1_ESM.docx]

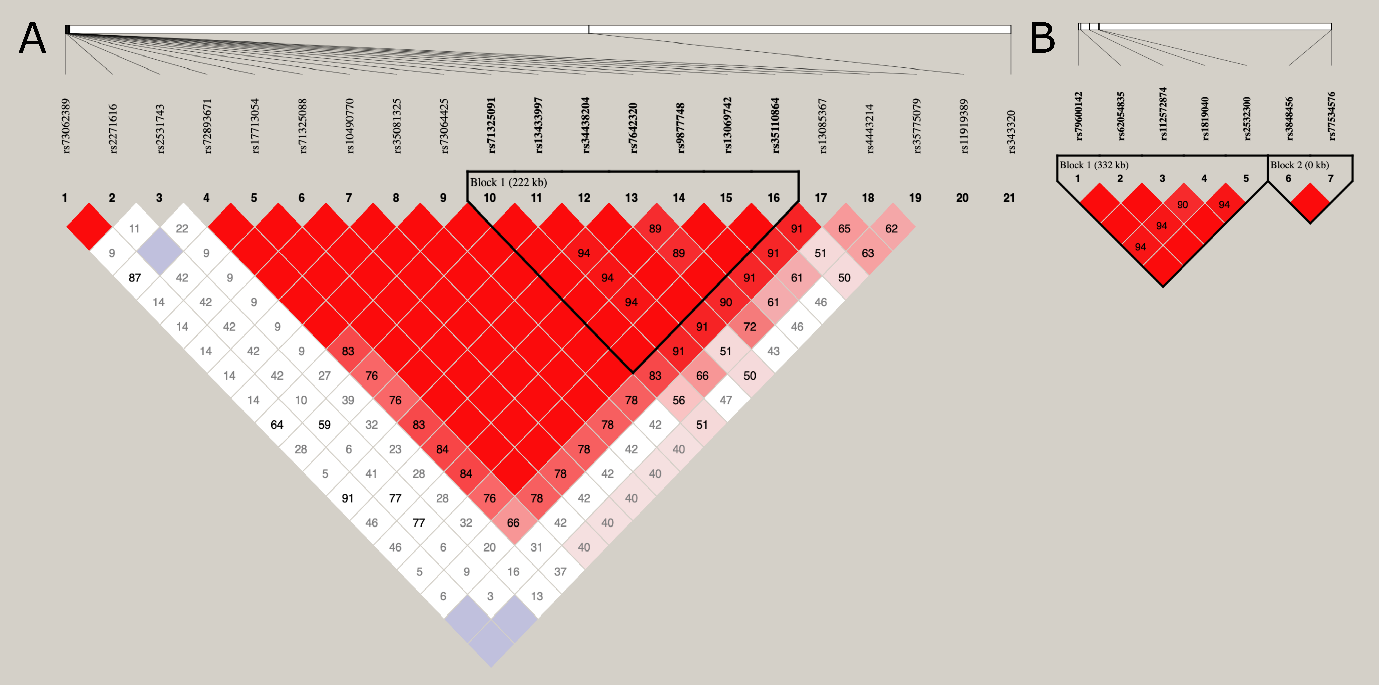


**Supplementary Figure 1.** Linkage disequilibrium analysis. Linkage disequilibrium between the SNPs located on chromosome 3 (A) and 17 (B) was analyzed by the Haploview 4.2 software. The squares show inside the value of D'. For the intense red squares in which numerical values are not included, D' = 1.
